# Unmet needs in the care of patients with neuromyelitis optica spectrum disorder and myelin oligodendrocyte glycoprotein antibody associated disease: insights from Germany

**DOI:** 10.1186/s42466-026-00503-6

**Published:** 2026-06-19

**Authors:** Katrin Giglhuber, Alix Bertrand, Clarissa Zappe, Ingo Kleiter, Ilya Ayzenberg, Achim Berthele

**Affiliations:** 1 Department of Neurology, TUM University Hospital, School of Medicine and Health, Technical University of Munich, Ismaninger Str. 22, Munich, 81675 Germany; 2 Queen Square MS Centre, Department of Neuroinflammation, UCL Queen Square Institute of Neurology, Faculty of Brain Sciences, University College London, London, UK; 3 Marianne-Strauss-Klinik, Berg, Germany; 4 Ruhr University Bochum, St. Josef Hospital, Department of Neurology, Bochum, Germany

**Keywords:** NMOSD, MOGAD, Healthcare provider survey, Patient care, Real-World setting

## Abstract

**Background:**

Neuromyelitis optica spectrum disorder (NMOSD) and myelin oligodendrocyte glycoprotein antibody-associated disease (MOGAD) are rare autoimmune disorders. Their true prevalence in Germany is unknown and can only be estimated from heterogeneous international data. Assuming 1–3 cases per 100,000 people for each disease suggests several thousand affected individuals nationwide, yet the German Neuromyelitis Optica Study Group (NEMOS) registry currently holds records of only about 1,300 patients seen in specialised centres. Numbers and care structures outside such facilities remain largely unknown. This survey aimed to assess the current state of NMOSD and MOGAD care in Germany, identify gaps, and inform future care strategies.

**Methods:**

An online questionnaire aimed at neurologists and neuropaediatricians was distributed via NEMOS, the German Neurological Society (DGN), the Professional Association of German Neurologists (BVDN), and the German Network for Research on Autoimmune Encephalitis (GENERATE) from March to May 2025. Questions addressed care structures, diagnostics, coding, treatment, guideline use, and practitioners’ needs.

**Results:**

A total of 104 physicians from all German federal states participated. Half worked in university hospitals, the remainder in other clinics and outpatient settings. Most were specialised in neuroimmunology (70.2%). Many reported an increase in patient numbers for NMOSD (55.8%) and MOGAD (77.4%). Diagnostic practices revealed significant inconsistencies: almost half of the respondents were unaware of their referral laboratory’s antibody assays, and ELISA remained in use despite clear recommendations for cell-based assays. ICD-10 coding varied widely. Off-label rituximab was most frequently used for first-line therapy of AQP4-antibody-positive NMOSD (69.6%), compared to satralizumab (57.1%), ravulizumab (55.4%) and inebilizumab (50.0%). AQP4-antibody-negative NMOSD was mainly treated with rituximab (87.0%). Also in MOGAD, rituximab was frequently used (by 58.9%), yet paediatricians preferred glucocorticoids and intravenous immunoglobulins. 69.6% initiated treatment for MOGAD after the first attack. Notably, 41.8% of physicians reported untreated NMOSD and 64.6% untreated MOGAD patients. Most respondents relied on national guidelines; 43.2% expressed a need for further education and patient information.

**Conclusions:**

Our findings highlight substantial heterogeneity in the diagnosis and treatment of NMOSD and MOGAD in Germany with potential implications for patient outcomes. This underscores the need for harmonised procedures and targeted educational resources to improve diagnostic reliability, treatment equity, and overall quality of care.

**Supplementary Information:**

The online version contains supplementary material available at 10.1186/s42466-026-00503-6.

## Background

Neuromyelitis optica spectrum disorder (NMOSD) and myelin oligodendrocyte glycoprotein (MOG)-antibody-associated disease (MOGAD) are two rare autoimmune conditions of the central nervous system, classified as orphan diseases [1]. The prevalence in Germany is unknown. Estimates based on heterogeneous international data suggest a prevalence of one to three cases per 100,000 people for each condition [2–4], thus affecting 2,000–5,000 individuals in Germany. The German Neuromyelitis Optica Study Group (NEMOS) registry currently holds records of approximately 1,300 NMOSD and MOGAD patients. This would indicate that fewer than half of the estimated patient population are managed in specialised centres. The care structures for the remaining patients, who are probably treated in general neurological practices or non-specialised clinics, remain largely unknown. Confirming or refuting these estimates is particularly difficult due to inconsistent reporting, a lack of harmonised records and epidemiological data in Germany, and uneven awareness.

Unlike other countries, Germany’s dual specialist system means care for rare diseases is provided in both hospital-affiliated and community outpatient settings. Consequently, practice-based neurologists can prescribe disease-specific therapies, rather than patients being referred exclusively to specialist centres. In this dual-track system, specialist networks play a crucial role in pooling expertise and raising awareness of rare diseases. One such network in Germany is NEMOS, which comprises more than 60 single centres, primarily university hospitals and centres with a neuroimmunological focus, that have joined due to their specific interest in the care of patients with NMOSD and MOGAD.

These conditions present multifaceted challenges for treating physicians. Beyond their rarity, both NMOSD and MOGAD require thorough laboratory and imaging diagnostics, present with unpredictable relapse patterns and the potential for severe disability, and necessitate highly individualised treatment decisions [5–8].

The current NMOSD diagnostic criteria were published eleven years ago [9]. Nevertheless, a recent Latin American survey revealed considerable variation in the application of these criteria among neurologists [10]. Regarding the diagnosis of MOGAD, the currently recommended criteria were proposed only three years ago and are therefore still very new [5]. This underscores the evolving nature of these nosological entities and the challenges clinicians face in keeping pace with diagnostic advances.

Both aquaporin-4 (AQP4)-antibody-positive NMOSD and MOG-antibody-associated disease are antibody-mediated, and antibody detection plays a crucial role in the diagnostic algorithm. Current international recommendations emphasise the use of cell-based assays (CBAs) for detecting AQP4-IgG and MOG-IgG, as these are more sensitive and specific than other methods, such as ELISA, western blot, or radioimmunoassay [11, 12]. For AQP4-IgG, fixed CBAs currently represent the most widely recognised and recommended assay, although live CBAs may further increase sensitivity and are likely to be incorporated into future diagnostic criteria [9, 13, 14]. For MOG-IgG, live CBAs are recommended and widely accepted as the reference standard [5, 15]. However, the extent to which these standards are implemented in routine care remains unclear.

The therapeutic landscape has also become increasingly complex. Since 2019, four monoclonal antibodies have received approval for the treatment of AQP4-antibody-positive NMOSD: eculizumab, satralizumab, inebilizumab, and ravulizumab [16–20]. While these offer targeted options, they also introduce significant cost disparities compared to the previously standard off-label agent, rituximab. In contrast to AQP4-antibody-positive NMOSD, no therapies are currently approved for AQP4-antibody-negative NMOSD and MOGAD, resulting in treatment strategies that are even more heterogeneous [21, 22].

Attempts to analyse shifts in treatment regimens or the general disease burden using statutory health insurance data are hindered substantially by inconsistent and suboptimal ICD-10 coding practices. Although the ICD-10 code *G36.0: Neuromyelitis optica [Devic syndrome]* is specific to NMOSD, it still might be underused in AQP4-antibody-negative NMOSD or misused in MOGAD due to its direct impact on treatment decisions and drug reimbursement. This non-specificity has significant implications for epidemiological research and clinical practice, potentially leading to patient misidentification or exclusion, an underestimation of disease prevalence, and an inability to accurately assess treatment patterns and effectiveness [23, 24].

In view of these uncertainties, there is an unmet need to better understand the “real-world” management of patients with NMOSD and MOGAD. The primary goal of this survey was to evaluate the current state of care in Germany across different institutional settings, to identify diagnostic and therapeutic gaps, and to inform future educational and healthcare strategies.

## Methods

### Questionnaire design

The questionnaire was designed and implemented using the online platform *LamaPoll* [25]. Depending on the individual response path, participants were presented with between 8 and 55 items across four main domains:

1. General care structures – place of work, neuroimmunological expertise and patient numbers,
2. Diagnosis – diagnostic procedures, antibody testing and ICD-10 coding,
3. Therapy – treatment approaches and monitoring, and
4. Resources – applied guidelines, training, patient support services and practitioners’ needs.

In addition, two case examples were included to explore expertise in diagnostic procedures. Several items allowed multiple responses, and some were conditionally displayed depending on prior answers. The full questionnaire can be found in the supplementary material (Suppl. 1).

### Data acquisition

The survey was conducted between March and May 2025. A link to the questionnaire was disseminated to physicians via NEMOS, the German Neurological Society (DGN), the Professional Association of German Neurologists (BVDN) and the German Network for Research on Autoimmune Encephalitis (GENERATE), with recipients encouraged to further share the invitation with colleagues. Responses were collected anonymously, and no personally identifiable information was obtained.

### Data analysis

Participants who indicated that they were neurologists or neuro-paediatricians (including both board-certified specialists and residents) and who had completed the initial two survey questions concerning the federal state and place of work were included in the analysis. Participants who did not complete the survey were identified at each question and excluded from subsequent analyses stepwise; the respective sample size is reported for each question. Analyses were based on the remaining valid sample size. Descriptive statistics were used to summarise participants’ demographics and survey responses, including mean, median, and range where appropriate. Free-text comments were reviewed, and relevant content was abstracted for analysis. Figures were generated using R (version 4.4.3).

## Results

### Study cohort

**Fig. 1:**
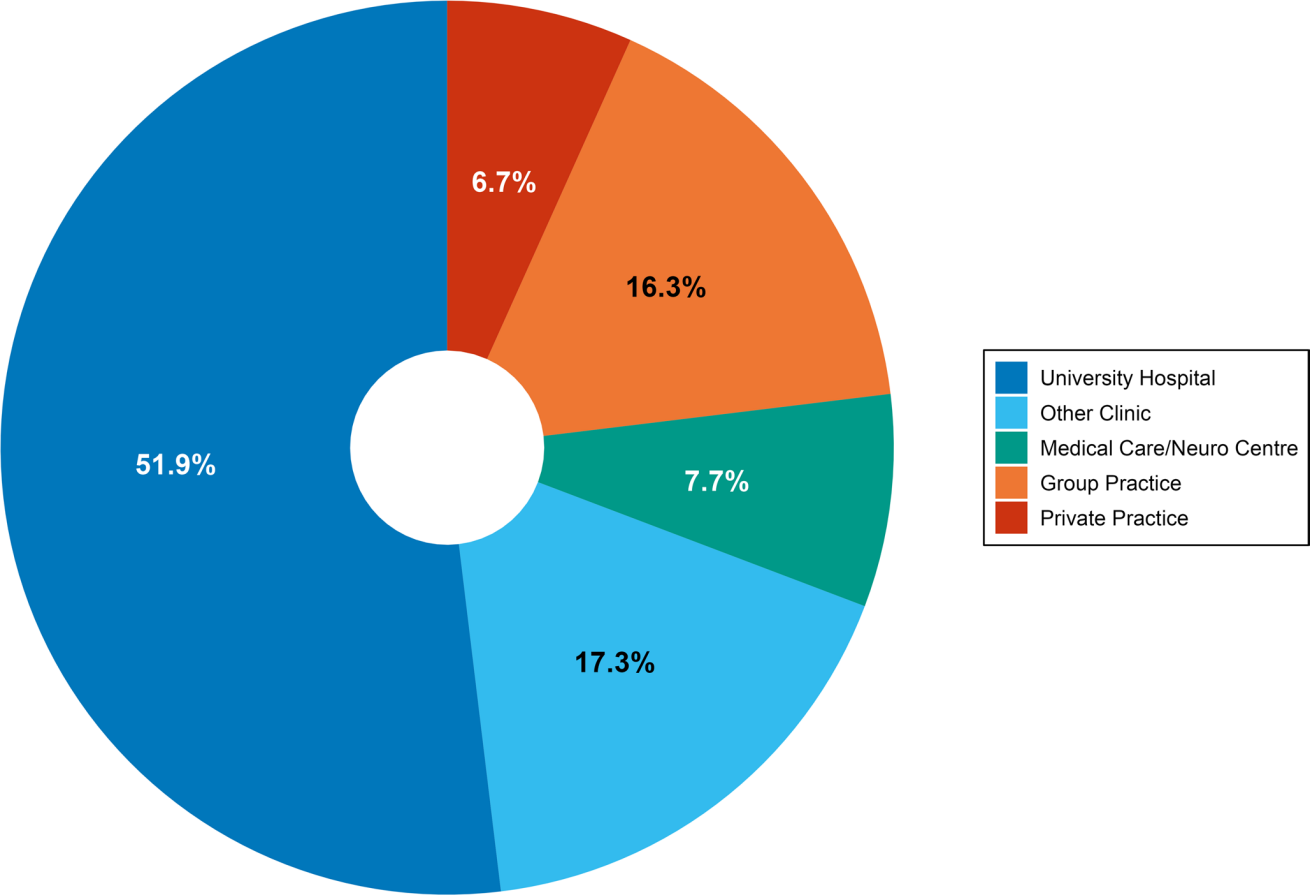
Participants’ affiliations. Donut chart showing the distribution of participants across different affiliations (% of total; *N* = 104). Each colour represents a different institution type: University Hospital, Other Clinic, Medical Care/Neuro Centre, Group Practice, and Private Practice (see legend)

Between March and July 2025, a total of 127 participants initiated the survey, of whom 104 were included in the analysis. They represented all 16 German federal states, with the highest proportions from North Rhine-Westphalia (21/104, 20.2%) and Bavaria (20/104, 19.2%), reflecting the number of inhabitants. Half of the respondents (54/104, 51.9%) were affiliated with university hospitals, 17.3% (18/104) with other clinics, and 30.8% (32/104) with individual or group practices and medical care centres (Fig. 1). A neuroimmunological focus was reported by 70.2% (73/104), half of which (39/73, 53.4%) were working at a NEMOS centre.

### General care structures

Of the respondents, 78.6% (77/98) reported treating NMOSD patients, of whom 88.3% (68/77) primarily managed adults and 11.4% (9/77) primarily children. Caseload varied considerably, with approximately one third (28/77, 36.4%) serving as primary physician for only 1–3 patients, while 18.2% (14/77) managed twenty patients or more. Most respondents (58/77, 75.9%) had been treating NMOSD patients throughout their clinical careers, while 24.7% (19/77) reported starting only after the emergence of treatment options. 55.8% (43/77) observed increasing NMOSD case numbers, primarily attributable to enhanced referrals and diagnostic awareness.

73.5% (72/98) reported treating MOGAD patients, with 84.7% (61/72) primarily managing adults and 15.3% (11/72) primarily children. Caseload distribution was comparable to NMOSD, with 41.7% (30/72) managing only 1–3 patients and 13.9% (10/72) managing twenty patients or more. (Neuro-)Paediatricians reported caring for proportionally higher numbers of MOGAD patients compared with adult neurologists. Regarding MOGAD patient numbers, a rising trend was reported by 76.4% (55/72).

Among the respondents, 42.9% (42/98) reported transferring NMOSD and MOGAD patients to specialised centres for a second opinion or for an annual study visit as part of the NEMOS national cohort study. Most non-referrers were already based at NEMOS centres (35/56, 62.5%) and university hospitals (45/56, 80.4%).

### Diagnosis

#### Laboratory testing

The vast majority of respondents (77/84, 91.7%) reported diagnosing NMOSD and MOGAD patients themselves. Amongst these, 44.0% (33/75) reported testing patients with any first manifestation of an autoimmune disorder of the central nervous system for antibodies against AQP4 and MOG, whereas the others restricted testing to patients with clinical or imaging features typical of NMOSD or MOGAD. The majority (64/75, 85.3%) routinely tested for both antibodies in parallel.

When asked about the referral laboratory used for antibody testing, 24.0% (18/75) of respondents were unaware which laboratory they used (“decision made by the routine laboratory”), and the other 76.0% (57/75) named a wide range of laboratories, ranging from major reference centres to in-house facilities (Table 1).

**Table 1:** Antibody testing

**Laboratory used for AQP4- and MOG-antibody testing**	**% (n/N)**
Do not know; samples sent to routine laboratory	24.0 (18/75)
Name a wide range of laboratories (free text)	76.0 (57/75)

**Know method used by referral laboratory for MOG-antibody testing**	
No	41.9 (31/74)
Yes	58.1 (43/74)
** If yes**,** which method**:	
Fixed CBA	51.2 (22/43)
Live CBA	37.2 (16/43)
ELISA	9.3 (4/43)
Stepwise: ELISA / fixed CBA / live CBA	2.3 (1/43)

**Know method used by referral laboratory for AQP4-antibody testing**	
No	46.7 (35/75)
Yes	53.3 (40/75)
** If yes**,** which method**:	
Fixed CBA	67.5 (27/40)
Live CBA	15.0 (6/40)
ELISA	10.0 (4/40)
Stepwise: ELISA / fixed CBA / live CBA	7.5 (3/40)

Questions and response options concerning antibody testing. Responses reported as percentage (n/N) of total. Aquaporin-4 (AQP4), myelin oligodendrocyte glycoprotein (MOG), cell-based assay (CBA)

Regarding the assay used at their referral laboratory for AQP4-antibody detection, half of the respondents (40/75, 53.3%) reported knowing the method, whereas the other half (35/75, 46.7%) did not. Amongst those aware of the assay, the majority used the recommended CBAs (fixed: 27/40 (67.5%), live: 6/40 (15.0%)), but nevertheless, 17.5% (7/40) reported using an ELISA or stepwise approaches that started with ELISA. Notably, of those unaware of the method, 85.7% (30/35) still diagnosed and treated NMOSD patients, and only about half (19/35, 54.3%) referred their patients to specialised centres for a second opinion.

For MOG-antibody testing, 58.1% (43/74) stated that they knew which assay was used. Among those practitioners, 51.2% (22/43) reported the use of a fixed CBA, 37.2% (16/43) a live CBA, and 11.6% (5/43) an ELISA or stepwise approach.

Almost all respondents (68/73, 93.2%) indicated that they would re-test in cases of persisting clinical suspicion of NMOSD or MOGAD in patients with initially negative results. When re-testing, 66.2% (45/68) used the same assay, 51.5% (35/68) also employed alternative serum testing approaches, and 36.8% (25/68) included cerebrospinal fluid testing. Most commonly, re-testing was performed after 3–6 months (47/68, 69.1%) or following a second attack (35/68, 51.5%).

#### ICD-10 coding

**Fig. 2:**
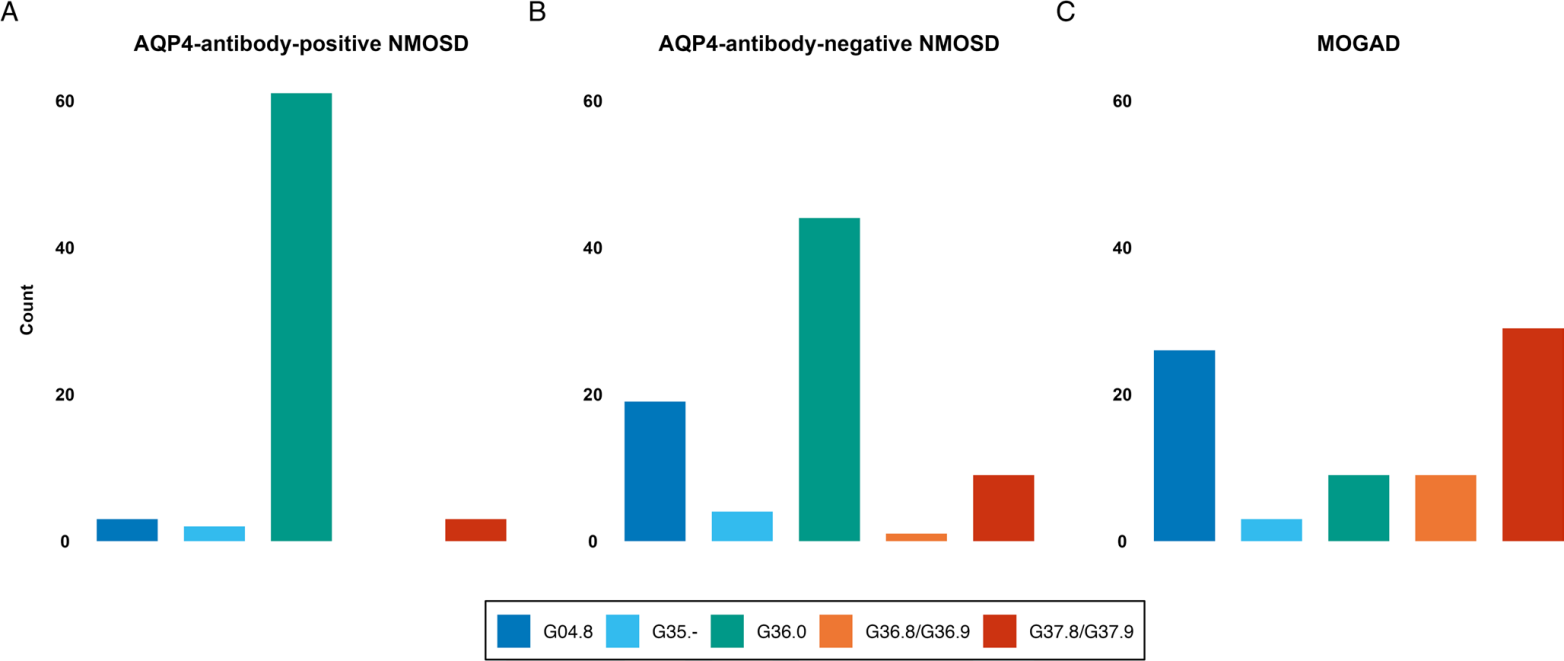
ICD-10 coding practices. ICD-10 code selections for AQP4-antibody-positive NMOSD **(A)**, AQP4-antibody-negative NMOSD **(B)**, and MOGAD **(C).** Bars indicate the absolute number of responses (of *N* = 77 respondents) with each colour representing a different ICD-10 code (see legend). G04.8: Other encephalitis, myelitis and encephalomyelitis, G35.-: Multiple sclerosis [Encephalomyelitis disseminata], G36.0: Neuromyelitis optica [Devic syndrome], G36.8/G36.9: Other specified acute disseminated demyelination/Acute disseminated demyelination, unspecified, G37.8/G37.9: Other specified demyelinating diseases of central nervous system/Demyelinating disease of central nervous system, unspecified. Multiple selections per participant were permitted. For AQP4-antibody-positive NMOSD no respondent selected G36.8/G36.9 (A, zero-height bar)

The majority of respondents (61/77, 79.2%, Fig. 2) coded AQP4-antibody-positive NMOSD as *G36.0: Neuromyelitis optica [Devic syndrome]* according to ICD-10. 18.2% (14/77) did not perform coding on their own.

Coding practices for AQP4-antibody-negative NMOSD were more heterogeneous: fewer than two-thirds (44/77, 57.1%) used *G36.0*, with many also applying.

- *G04.8: Other encephalitis*,* myelitis and encephalomyelitis*,
- *G35.-: Multiple sclerosis [Encephalomyelitis disseminata]*,
- *G36.8/G36.9: Other specified acute disseminated demyelination/Acute disseminated demyelination*,* unspecified*, or.
- *G37.8/G37.9: Other specified demyelinating diseases of central nervous system/Demyelinating disease of central nervous system*,* unspecified*.

For MOGAD, the codes *G04.8* and *G37.8/G37.9* were used by around one third of respondents (26/77 and 29/77). Fewer applied *G36.0*, * G35.- and G36.8/G36.9.*

### Therapy

Of 77 respondents, 58 (75.3%) reported initiating or switching therapies themselves, all of whom were able to administer intravenous treatments.

#### AQP4-antibody-positive NMOSD

**Fig. 3:**
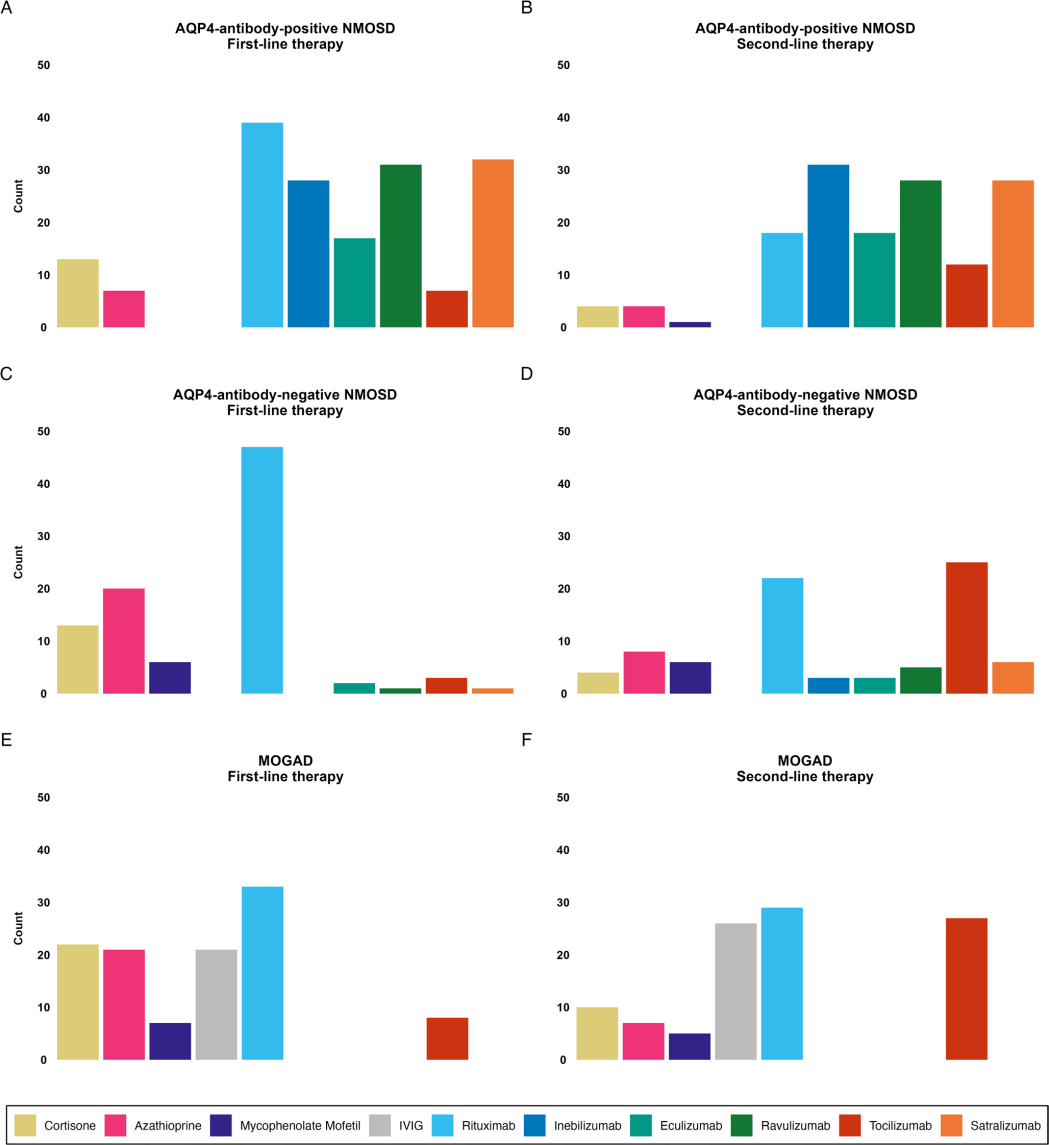
Immunotherapy choices. Drug selections for first-line and second-line treatment in AQP4-antibody-positive NMOSD **(A**,** B)**, AQP4-antibody-negative NMOSD **(C**,** D**), and MOGAD **(E**,** F).** Bars represent absolute numbers of responses (of *N* = 56 respondents), with each colour representing a different drug: Cortisone, Azathioprine, Mycophenolate Mofetil, IVIG, Rituximab, Inebilizumab, Eculizumab, Ravulizumab, Tocilizumab, and Satralizumab (see legend). Multiple selections per participant were permitted. Zero-height bars represent drugs with no reported use

For first-line treatment of AQP4-antibody-positive NMOSD, 69.6% (39/56) reported using rituximab, 57.1% (32/56) satralizumab, 55.4% (31/56) ravulizumab, 50.0% (28/56) inebilizumab, 30.4% (17/56) eculizumab and 23.2% (13/56) oral glucocorticoids (Fig. 3A). When asked for their preferred therapy, 13 of 36 respondents (36.1%) named rituximab.

For second-line therapy in AQP4-antibody-positive NMOSD, inebilizumab was preferred (31/52, 59.6%), followed by ravulizumab and satralizumab (each by 28/52, 51.9%, Fig. 3B). Escalation of treatment was reported to be required in an average of 15.4% of cases.

With respect to rituximab, 77.2% (44/57) reported using it off-label in AQP4-antibody-positive NMOSD when reimbursement was granted, with most of them applying for coverage themselves.

Regarding complement inhibitors, most respondents (40/53, 75.5%) initiated therapy with ravulizumab as their routine first-line option. In addition, 28.1% (16/57) indicated switching all patients previously treated with eculizumab to ravulizumab.

#### AQP4-antibody-negative NMOSD

For the first-line treatment of AQP4-antibody-negative NMOSD, the majority reported using rituximab (47/54, 87.0%), followed by azathioprine (20/54, 37.0%) and oral glucocorticoids (13/54, 24.1%, Fig. 3C). All respondents used rituximab off-label for AQP4-antibody-negative NMOSD when reimbursement was granted.

Escalation therapy was reported to be required in an average of 16.9% of cases. For these patients, tocilizumab was the most used second-line agent (25/50, 50.0%), followed by rituximab (22/50, 44.0%, Fig. 3D).

While 58.2% (39/67) reported treating all their (AQP4-antibody-positive and -negative) NMOSD patients, 41.8% (28/67) also had untreated cases, of which one-third (10/28, 35.7%) reported exclusively AQP4-antibody-negative untreated patients.

#### MOGAD

Around two-thirds of respondents (39/56, 69.6%) initiated treatment for MOGAD after the first attack, regardless of whether they were primarily treating children (6/9, 66.7%) or adults (33/45, 73.3%). Several free-text responses reflected individualized treatment approaches depending on attack severity, recovery, and antibody titres.

For MOGAD first-line treatment, rituximab (33/56, 58.9%) was most frequently used, followed by oral glucocorticoids (22/56, 39.3%), intravenous immunoglobulins (IVIG), and azathioprine (each by 21/56, 37.5%, Fig. 3E). For MOGAD second-line therapy, practitioners applied rituximab (29/56, 51.8%), tocilizumab (27/56, 48.2%), and IVIG (26/56, 46.4%, Fig. 3F).

Among nine respondents who primarily treated paediatric MOGAD patients, oral glucocorticoids and IVIG were the preferred first-line treatments (applied each by 4/9, 44.4%), while IVIG (5/9, 55.6%) and rituximab (4/9, 44.4%) dominated second-line therapy.

64.6% of respondents (42/65) had MOGAD patients without current therapy, whereas the other 35.4% (23/65) treated all their patients.

#### Monitoring

The majority of respondents (66/75, 88.0%) used the Expanded Disability Status Scale (EDSS) for clinical monitoring. In addition, 64.0% (48/75) assessed visual function, and 53.3% (40/75) walking ability. Paediatricians reported using tests adapted to the child’s developmental stage.

Regarding MR diagnostics, 18.1% (13/72) reported performing imaging only at diagnosis and during attacks, whereas 81.9% (59/72) indicated routine imaging, most commonly on an annual basis.

### Resources

To guide treatment decisions for NMOSD and MOGAD, most respondents (48/56, 85.7%) indicated relying on the national guidelines of the DGN, followed by recommendations of NEMOS (46/56, 82.1%) and presentations or discussions at specialist conferences (44/56, 78.6%).

Awareness of patient self-help organizations was limited, with only 41.9% (31/74) reporting knowledge of such resources.

When asked about additional needs to support the diagnosis and management of NMOSD and MOGAD, 43.2% (38/88) expressed a demand for further resources. The most frequently mentioned priorities were education and training (particularly online formats, and content tailored to paediatric NMOSD/MOGAD), patient information materials, and structured guidance, including updated recommendations, improved networking, and greater laboratory transparency.

### Case examples

Case #1 concerned a 39-year-old female patient with a medical history of optic neuritis and myelitis, who was now exhibiting signs of paraparesis and bowel dysfunction, with longitudinal, extensive spinal cord involvement on MRI (Table 2). Most respondents (63/67, 94.0%) correctly identified that this patient met dissemination in space criteria for NMOSD diagnosis and recognised that the patient met the IPND 2015 diagnostic criteria even in the case of negative antibody findings (62/65, 95.4%). However, divergent opinions emerged when respondents were asked to consider a modified scenario without a history of optic neuritis but with prolonged visual evoked potentials, in which case 40.3% (27/67) erroneously deemed this sufficient to meet the criteria.

**Table 2:** **Case #1**

**CASE VIGNETTE (1 of 2)** • 39-year-old woman • History of optic neuritis in the right eye (confirmed by ophthalmologist); history of myelitis with residual deficits • Current symptoms: paraparesis of the legs, bowel dysfunction • Brain MRI: unremarkable • Spinal cord MRI: T2-hyperintense lesion at level T1-T8 with contrast enhancement	
**Does this patient meet dissemination in space criteria for NMOSD diagnosis (according to the IPND 2015 Criteria)?**	**% (n/N)**
Yes	94.0 (63/67)
No	6.0 (4/67)
**If the prior optic neuritis were absent**,** but visual evoked potentials of the right eye were prolonged (despite absence of clinical symptoms in that eye)**,** would the NMOSD diagnostic criteria be met?**	
Yes	40.3 (27/67)
No	59.7 (40/67)
**Based on the above information**,** does this patient meet the criteria for NMOSD diagnosis (according to the IPND 2015 Criteria) if she is AQP4-IgG-seronegative?**	
No, this patient does not have a core clinical characteristic of NMOSD.	0
No, although this patient presents with a core clinical characteristic of NMOSD, since she is AQP4-IgG-seronegative.	3.1 (2/65)
Yes, provided there is no better explanation, because she has a core clinical characteristic of NMOSD, a history of other core clinical characteristics, and the required supporting MRI findings.	95.4 (62/65)
No, this patient presents with a core clinical characteristic of NMOSD, but meets neither dissemination in space nor dissemination in time criteria and therefore does not have NMOSD.	1.5 (1/65)

Description of Case #1, questions and response options. Responses reported as percentage (n/N) of total. Correct response options are underlined

Case #2 involved a 28-year-old female patient with a history of bilateral optic neuritis without residuals (Table 3). The patient subsequently presented with new vision loss and pain in the right eye, accompanied by bilateral longitudinally extensive optic nerve involvement on MRI. Almost all respondents (63/65, 96.9%) correctly concluded that MOG antibody testing should be performed alongside AQP4 antibody testing for this clinical presentation. Given borderline-positive MOG-IgG serology (titre 1:32) and negative AQP4-IgG, the majority (51/64, 79.7%) of neurologists diagnosed the patient with MOGAD in line with the current criteria [5].

**Table 3:** Case #2

**CASE VIGNETTE (2 of 2)** • 28-year-old woman • History of bilateral optic neuritis without residual deficits • Current symptoms: recurrent visual deterioration in the right eye, pronounced pain with eye movement • Brain MRI: bilateral longitudinally extensive T2-hyperintense optic nerves, otherwise unremarkable • Spinal cord MRI: unremarkable	
**Would you test this patient for MOG antibodies?**	**% (n**/**N)**
Yes, because bilateral optic neuritis excludes the diagnosis of multiple sclerosis.	0
No, the absence of residual deficits from prior optic neuritis suggests the diagnosis of multiple sclerosis.	0
No, this clinical presentation is more typical of NMOSD and I only test for AQP4 antibodies.	0
Yes, in combination with testing for AQP4 antibodies.	96.9 (63/65)
No, because a diagnosis of MOGAD could not be made due to the absence of dissemination in space.	3.1 (2/65)
**Does this patient meet the current diagnostic criteria for MOGAD if the MOG-IgG titre is only borderline positive with 1:32?**	
No, because she has only one core clinical event of MOGAD.	1.6 (1/64)
No, although she has a core clinical event of MOGAD, since she lacks supporting clinical or radiologic features.	14.1 (9/64)
Yes, provided she is AQP4-IgG-seronegative and alternative diagnoses have been excluded, because she has a core clinical characteristic of MOGAD with required supporting MRI findings.	79.7 (51/64)
No, she demonstrates a core clinical event with supporting MRI findings of NMOSD, so MOGAD diagnostic criteria should not be applied.	4.7 (3/64)

Description of Case #2, questions and response options. Responses reported as percentage (n/N) of total. Correct response options are underlined

## Discussion

The results of our survey on the current management of NMOSD and MOGAD in Germany exemplify the shortcomings in the handling of rare neurological diseases: Despite the availability of clear diagnostic criteria and the emergence of new medications, substantial disparities and unmet needs persist in routine clinical practice. Moreover, it emphasizes once again the challenges of accurately depicting the real world of rare disease management in the face of a paucity of rapport, consistency and knowledge.

### Responder characteristics reflecting care structures and specialist engagement

There are around 6,700 ambulatory neurological practitioners in Germany and the number of DGN members exceeds 13,500 [26, 27]. A total of 127 individuals responded to the survey advertisement. Approximately two-thirds of the respondents practised in hospitals and one-third in the outpatient sector, which closely reflects the overall distribution of neurologists in Germany [28]. While a notable proportion managed small patient cohorts, suggesting the inclusion of practitioners outside large academic centres, a majority reported a neuroimmunological focus. Consequently, we must assume a selection bias towards particularly engaged specialists. Only a small proportion of our respondents who practise outside reference centres did not refer their patients to specialised centres, yet many other practitioners without referral networks may not have been available for the survey or simply never heard about it. It is impossible to quantify the magnitude of this selection bias, but it is likely to be substantial. This reflects the inherent limitations of surveying care structures of low-prevalence diseases in a real-world setting. Two further limitations were introduced by the anonymous study design. Firstly, multiple participants from the same institution could not be ruled out, meaning that some answers may have reflected a collective decision-making for identical patients. Secondly, without data on years of clinical practice, we could not assess the influence of experience levels on diagnostic and treatment approaches.

### The antibody-testing problem

The most striking finding of our study was the low knowledge and precision regarding antibody testing. The NMOSD and MOGAD communities rigorously advocate for CBA, increasingly supported by studies suggesting even superior sensitivity with live versus fixed cells [12, 14, 15]. Whilst countries such as France maintain reference networks with centralised antibody testing (the NOMADMUS network), Germany so far lacks a standardised diagnostic infrastructure. Multiple laboratories offer AQP4- and MOG-IgG testing using heterogeneous assay methods without standardisation or quality benchmarks. Consequently, guideline recommendations remain unevenly adopted across centres. Some respondents referred samples to their “routine laboratory” without knowing whether or where they were transferred onward or which method was used. Of those who were aware of their referral laboratory, only half could specify the assays used, and a concerning minority still reported using ELISA. As antibody status is crucial for diagnosis, treatment selection and prognosis in NMOSD and MOGAD, inaccurate results from suboptimal testing methods can compromise the entire therapeutic process, potentially from the very first step [29]. This raises another difficult-to-investigate question: How many patients with NMOSD and MOGAD may be misclassified and consequently receive incorrect treatment due to diagnostic error?

### Coding as the blind spot of health services research

The ICD-10 coding analysis revealed a differentiated, but ultimately problematic picture. In most cases, AQP4-antibody-positive NMOSD was coded accurately. However, AQP4-antibody-negative NMOSD was coded correctly far less frequently, and for MOGAD, for which there is no specific ICD-10 code yet, practitioners employed entirely heterogeneous coding strategies. This is more than just an administrative inconvenience. Claims-based analyses, increasingly used as the basis for health services research and cost-effectiveness evaluations, depend entirely on coding accuracy. If coding for these diseases is already so inconsistent, the epidemiological and economic analyses derived from such data will be systematically biased.

### Application of diagnostic criteria

The short case examples showed that practitioners generally applied the current NMOSD diagnostic criteria correctly - although specific scenarios, such as subclinical latency delays in visually evoked potentials, can seemingly lead to misinterpretation, as was found in the aforementioned survey by the Latin American colleagues [10]. Diagnosing MOGAD with borderline antibody titres proved substantially more challenging. This is unsurprising, given that MOGAD is a more recent nosological entity than NMOSD, and the diagnostic criteria are less well established [5].

### Rock-solid Rituximab

Rituximab remains a solid rock in the therapy landscape in Germany across all three conditions, which is significant from both clinical and economic perspectives. In AQP4-antibody-negative NMOSD, rituximab is widely used. In AQP4-antibody-positive NMOSD, it remains a preferred treatment option despite the introduction of new and approved medications. This is unsurprising, as it aligns with earlier as well as recent analyses of the NEMOS registry data, and implicitly validates the efficacy and safety of this off-label drug over a period of around two decades [30–34]. For MOGAD, however, the situation is more nuanced, revealing an instructive gap between guideline recommendations and clinical practice. Recent data suggest that MOGAD patients treated with rituximab experience less favourable outcomes compared to those receiving IVIG [35], with rituximab showing similar efficacy to azathioprine [36], but this is not yet reflected in our real-world data. The persistent predominance of rituximab in clinical practice also highlights a critical question in the context of the orphan drug debate: if affordable, established off-label therapies are effective, then the added value of new, more expensive medications must be demonstrated not only in clinical trials but also under real-world conditions. However, prospective, randomised, head-to-head comparisons between new drugs and rituximab for the treatment of NMOSD are still pending. A similar scenario is emerging for MOGAD: after years of using established immunotherapies off-label, major clinical trials are now underway, and the METEORID trial on satralizumab recently reported positive results [37, 38]. In this shifting landscape, our findings provide a baseline to evaluate the future translation of emerging treatments into clinical practice.

### Future implications and suggestions

Taken together, our findings reveal a clear need for education, standardisation, and harmonisation of diagnostic and therapeutic procedures across the neurological community. Following our real-world insights from Germany, we suggest:

- Building awareness of state-of-the-art antibody-testing in laboratories.
- Incorporating guidance on the relevance of correct ICD-10-coding into guidelines.
- Establishing educational and training programs, including open case discussion rounds, to keep practitioners up-to-date and encourage cooperation.

Furthermore, our results indicate a systemic gap that extends far beyond NMOSD and MOGAD [39, 40]. While increasing knowledge and subspecialisation in neurology undoubtedly improves expert care for complex disorders, it also risks fragmenting general patient pathways. Reference networks such as NEMOS and NOMADMUS are essential in bridging the gap between general neurologists and highly specialised centres. Crucially, these issues cannot be resolved by medical disciplines alone; they require targeted system-level investments, such as integrated digital referral platforms or unified diagnostic registries, and a rethinking of ‘innovation’ in the management of rare diseases.

## Conclusions

Although our findings are based on a German cohort, they highlight universal challenges in managing rare neurological conditions. They emphasise the urgent need for standardised laboratory procedures, harmonised ICD-10 coding, closer collaboration between specialist centres, general neurological practitioners and laboratories, and targeted educational initiatives. Implementing these measures will not only improve the care for NMOSD and MOGAD in Germany but can also serve as a blueprint for enhancing consistency and quality of care for rare neurological diseases globally.

## Supplementary Information

Supplementary material 1.

## Data Availability

The dataset generated and/or analysed during the current study are not publicly available due to local regulations concerning data protection but are available from the corresponding author on reasonable request.

## Abbreviations

AQP4: Aquaporin-4
CBA: Cell-based assay
EDSS: Expanded disability status scale
GENERATE: German network for research on autoimmune encephalitis
DGN: German Neurological Society
IVIG: Intravenous immunoglobulin
MOG: Myelin oligodendrocyte glycoprotein
MOGAD: Myelin oligodendrocyte glycoprotein antibody-associated disease
NMOSD: Neuromyelitis optica spectrum disorder
NEMOS: Neuromyelitis optica study group
BVDN: Professional association of German neurologists
