# Supplementary material for: Unmet needs in the care of patients with neuromyelitis optica spectrum disorder and myelin oligodendrocyte glycoprotein antibody associated disease: insights from Germany
Source: Neurol Res Pract. 2026 Jun 19;8(1):48. doi: 10.1186/s42466-026-00503-6 (PMC13282859; doi:10.1186/s42466-026-00503-6)
Supplement: Supplementary file 1 — Supplementary material 1. [file 42466_2026_503_MOESM1_ESM.docx]

**Supplementary Appendix 1: Questionnaire (English Translation).**

**1. In which federal state do you practice?** (Single choice)
( ) Baden-Württemberg
( ) Bavaria
( ) Berlin
( ) Brandenburg
( ) Bremen
( ) Hamburg
( ) Hesse
( ) Mecklenburg-Western Pomerania
( ) Lower Saxony
( ) North Rhine-Westphalia
( ) Rhineland-Palatinate
( ) Saarland
( ) Saxony
( ) Saxony-Anhalt
( ) Schleswig-Holstein
( ) Thuringia
( ) Other region: [Free text]

**2. In which setting do you see neurological patients?** (Multiple choice)
☐ University hospital
☐ Other Clinic
☐ Medical Care/Neuro Centre
☐ Group practice
☐ Private practice
☐ I am not a neurologist, but rather: [Free text]
☐ Other setting: [Free text]

**3. Do you have a focus on neuroimmunology?** (Single choice)
( ) Yes
( ) No

**3a. If yes: Where are you active?** (Multiple choice)
☐ University outpatient clinic
☐ Centre of the Neuromyelitis Optica Study Group (NEMOS)
☐ §116b-outpatient clinic/ASV-MS
☐ DMSG-certified centre
☐ Authorised outpatient clinic
☐ I work only in an inpatient setting.

**4. Do you treat patients with NMOSD?** (Single choice)
( ) Yes
( ) No

**4a. If yes: Do you primarily treat adults or children and adolescents?** (Single choice)
( ) Primarily adults
( ) Primarily children and adolescents

**4b. If yes: For how many NMOSD patients are you the primary treating physician?** (Single choice)
( ) 0
( ) 1-3
( ) 4-9
( ) 10-19
( ) ≥20

**4c. If yes: When did you start treating patients with NMOSD?** (Single choice)
( ) Always
( ) Since pharmacological treatment options became available
( ) Since approved medications became available

**4d. If yes: Is the number of NMOSD patients you treat changing?** (Multiple choice)

☐ Yes, it is increasing because colleagues refer patients to me.
☐ Yes, it is increasing because the diagnosis is detected more frequently.
☐ Yes, it is decreasing because more colleagues feel confident to treat these patients.
☐ Yes, it is decreasing because these patients are increasingly being cared for in specialised clinics.
☐ No, it remains stable.

**5. Do you treat patients with MOGAD?** (Single choice)
( ) Yes
( ) No

**5a. If yes: Do you primarily treat adults or children and adolescents?** (Single choice)
( ) Primarily adults
( ) Primarily children and adolescents

**5b. If yes: For how many MOGAD patients are you the primary treating physician?** (Single choice)
( ) 0
( ) 1-3
( ) 4-9
( ) 10-19
( ) ≥20

**5c. If yes: Is the number of MOGAD patients you treat changing?** (Multiple choice)
☐ Yes, it is increasing because colleagues refer patients to me.
☐ Yes, it is increasing because the diagnosis is detected more frequently.
☐ Yes, it is decreasing because more colleagues feel confident to treat these patients.
☐ Yes, it is decreasing because these patients are increasingly being cared for in specialised clinics.
☐ No, it remains stable.

**6. Do you refer patients with (suspected) NMOSD or MOGAD to specialised centres?** (Single choice)
( ) Yes
( ) No

**6a. If yes: When/why?** (Multiple choice)
☐ For diagnostic workup
☐ For initial therapy consultation
☐ For joint evaluation or second opinion
☐ For co-management or annual NEMOS centre visit

**7. How did you learn about this questionnaire?** (Multiple choice)
☐ German Neurological Society (DGN)
☐ Professional Association of German Neurologists (BVDN)
☐ Neuromyelitis Optica Study Group (NEMOS)
☐ Other: [Free text]

**8. Do you diagnose patients with NMOSD and MOGAD yourself?** (Single choice)
( ) Yes
( ) No

**9. Which ICD-10 coding(s) do you use when diagnosing AQP4-antibody-positive NMOSD?** (Multiple choice)
☐ G04.8: Other specified encephalitis, myelitis and encephalomyelitis
☐ G35.-: Multiple sclerosis
☐ G36.0: Neuromyelitis optica [Devic syndrome]
☐ G36.8/G36.9: Acute disseminated demyelination
☐ G37.8/G37.9: Demyelinating disease of the central nervous system
☐ None, I do not code myself.
☐ Other: [Free text]

**10. Which ICD-10 coding(s) do you use when diagnosing AQP4-antibody-negative NMOSD?** (Multiple choice)
☐ G04.8: Other specified encephalitis, myelitis and encephalomyelitis
☐ G35.-: Multiple sclerosis
☐ G36.0: Neuromyelitis optica [Devic syndrome]
☐ G36.8/G36.9: Acute disseminated demyelination
☐ G37.8/G37.9: Demyelinating disease of the central nervous system
☐ None, I do not code myself.
☐ Other: [Free text]

**11. Has your coding behaviour changed since the approval of specific medications for treating AQP4-antibody-positive NMOSD?** (Single choice)
( ) No

( ) Yes, I use G36.0 more frequently.
( ) Yes, other: [Free text]

**12. Which ICD-10 coding(s) do you use when diagnosing MOGAD?** (Multiple choice)
☐ G04.8: Other specified encephalitis, myelitis and encephalomyelitis
☐ G35.-: Multiple sclerosis
☐ G36.0: Neuromyelitis optica [Devic syndrome]
☐ G36.8/G36.9: Acute disseminated demyelination
☐ G37.8/G37.9: Demyelinating disease of the central nervous system
☐ None, I do not code myself.
☐ Other: [Free text]

**13. Which patients do you test for aquaporin-4 (AQP4) and/or myelin oligodendrocyte glycoprotein (MOG) antibodies upon new diagnosis of a chronic inflammatory CNS disease?** (Single choice)
( ) All patients
( ) Only patients with clinical and/or MRI findings typical for NMOSD or MOGAD
( ) None

**14. Which antibodies do you test for?** (Single choice)
( ) Always together: I routinely test for AQP4 and MOG antibodies simultaneously.
( ) Tailored to suspected diagnosis: I test for AQP4 or MOG antibodies based on clinical presentation (not routinely simultaneously).
( ) Only AQP4: I test exclusively for AQP4 antibodies, not for MOG.
( ) Only MOG: I test exclusively for MOG antibodies, not for AQP4.
( ) Other: [Free text]

**15. To which laboratory do you send your samples?** *Please provide separate responses for AQP4 and MOG antibodies, if applicable.* (Multiple choice)
☐ For AQP4 antibodies: [Free text]
☐ For MOG antibodies: [Free text]
☐ I do not know; this is decided by my routine laboratory.

**16. Do you know which test method/assay your laboratory uses to detect AQP4 antibodies?** (Single choice)
( ) Yes
( ) No

**16a. If yes: Which method?** (Single choice)
( ) Fixed cell-based assay
( ) Live cell-based assay
( ) ELISA
( ) Stepwise: ELISA / fixed cell-based assay / live cell-based assay

**17. Do you know which test method/assay your laboratory uses to detect MOG antibodies?** (Single choice)
( ) Yes
( ) No

**17a. If yes: Which method?** (Single choice)
( ) Fixed cell-based assay
( ) Live cell-based assay
( ) ELISA
( ) Stepwise: ELISA / fixed cell-based assay / live cell-based assay

**18. Do you repeat testing for MOG or AQP4 antibodies if the initial result is negative but there is high clinical suspicion?** (Single choice)
( ) Yes
( ) No

**18a. If yes: Where?** (Multiple choice)
☐ In blood with the same test
☐ In blood with a different test
☐ In cerebrospinal fluid

**18b. If yes: When?** (Multiple choice)
☐ Immediately
☐ After ___ months: [Numerical entry]
☐ At/after the next attack

**19. When do you perform MRI scans?** (Multiple choice)
☐ At time of diagnosis
☐ Upon attack symptoms
☐ Regularly, every ___ months: [Numerical entry]

**20. Do you initiate or switch immunotherapy in patients with NMOSD or MOGAD on your own?** (Single choice)
( ) Yes
( ) No

**20a. If yes: Are you able to administer intravenous therapies?** (Single choice)
( ) Yes

( ) No. This is why I do not treat NMOSD/MOGAD patients.
( ) No, but I can offer this through a collaboration.
( ) No.

**20b. If yes: Which medications do you administer?** (Multiple choice)
☐ Eculizumab
☐ Inebilizumab
☐ Intravenous immunoglobulins
☐ Intravenous corticosteroids
☐ Ravulizumab
☐ Rituximab
☐ Satralizumab
☐ Tocilizumab
☐ Other: [Free text]

**21. Which medications do you use as the primary treating physician for first-line therapy in AQP4-antibody-positive NMOSD?** (Multiple choice)
☐ Azathioprine
☐ Eculizumab
☐ Inebilizumab
☐ Oral corticosteroids
☐ Mycophenolate mofetil
☐ Ravulizumab
☐ Rituximab
☐ Satralizumab
☐ Tocilizumab
☐ Other: [Free text]

**21a. Do you have a favourite?**
[Free text]

**22. Which medications do you use for AQP4-antibody-positive NMOSD after failure of first-line therapy?** (Multiple choice)
☐ Azathioprine
☐ Eculizumab
☐ Inebilizumab
☐ Oral corticosteroids
☐ Mycophenolate mofetil
☐ Ravulizumab
☐ Rituximab
☐ Satralizumab
☐ Tocilizumab
☐ Other: [Free text]

**22a. In approximately what percentage of your patients is a second-line therapy necessary?** (Slider)
[0% ——— 100%]

**23. Do you use rituximab off-label for treating AQP4-antibody-positive NMOSD?** (Multiple choice)
☐ Yes, if reimbursement by the health insurance is approved.
☐ Yes, and I apply for reimbursement myself.
☐ No, I do not initiate therapy with rituximab.
☐ No, I switch patients to an approved medication.
☐ No, the approved medications are more effective.

**24. How do you use complement inhibitors?** (Multiple choice)
☐ I still always start with eculizumab.
☐ I now always start with ravulizumab.
☐ I switch all patients being treated with eculizumab to ravulizumab.
☐ I switch patients being treated with eculizumab to ravulizumab only upon explicit patient request.
☐ I have already switched patients from ravulizumab back to eculizumab.

**25. Which medications do you use as the primary treating physician for first-line therapy in AQP4-antibody-negative NMOSD?** (Multiple choice)
☐ Azathioprine
☐ Eculizumab
☐ Inebilizumab
☐ Oral corticosteroids
☐ Mycophenolate mofetil
☐ Ravulizumab
☐ Rituximab
☐ Satralizumab
☐ Tocilizumab
☐ Other: [Free text]

**26. Which medications do you use for AQP4-antibody-negative NMOSD after failure of first-line therapy?** (Multiple choice)
☐ Azathioprine
☐ Eculizumab
☐ Inebilizumab
☐ Oral corticosteroids
☐ Mycophenolate mofetil
☐ Ravulizumab
☐ Rituximab
☐ Satralizumab
☐ Tocilizumab
☐ Other: [Free text]

**26a. In approximately what percentage of your patients is a second-line therapy necessary?** (Slider)
[0% ——— 100%]

**27. Do you use rituximab off-label for treating AQP4-antibody-negative NMOSD?** (Single choice)
( ) Yes, if reimbursement by the health insurance is approved.
( ) Yes, and I apply for reimbursement myself.
( ) No.

**28. Do you have NMOSD patients who do not receive immunotherapy?** (Single choice)
( ) Yes

( ) Yes, but only AQP4-anitbody-negative patients
( ) No

**29. Which medications do you use as the primary treating physician for first-line therapy in MOGAD?** (Multiple choice)
☐ Azathioprine
☐ Intravenous immunoglobulins
☐ Oral corticosteroids
☐ Mycophenolate mofetil
☐ Rituximab
☐ Tocilizumab
☐ Other: [Free text]

**30. Which medications do you use for MOGAD after failure of first-line therapy?** (Multiple choice)
☐ Azathioprine
☐ Intravenous immunoglobulins
☐ Oral corticosteroids
☐ Mycophenolate mofetil
☐ Rituximab
☐ Tocilizumab
☐ Other: [Free text]

**31. When do you initiate therapy in patients with MOGAD?** (Multiple choice)
☐ After the first attack
☐ After the second attack
☐ After confirmed antibody detection after ___ months: [Numerical entry]
☐ Other: [Free text]

**32. Do you have MOGAD patients who do not receive immunotherapy?** (Multiple choice)
☐ Yes
☐ No
☐ ...and I have patients whom I treat with immunotherapies approved for multiple sclerosis, as I now consider this diagnosis more likely.

**33. How do you monitor patients with NMOSD and MOGAD clinically?** (Multiple choice)
☐ Expanded Disability Status Scale (EDSS)
☐ Multiple sclerosis functional composite (MSFC)
☐ Visual acuity testing
☐ Ambulation testing
☐ National Eye Institute Visual Function Questionnaire (NEIVFQ)
☐ Other: [Free text]

**34. Based on which information sources do you treat patients with NMOSD and MOGAD?** (Multiple choice)
☐ German Neurological Society (DGN) guidelines
☐ NEMOS recommendations
☐ Lectures and discussions at specialist conferences
☐ Articles in specialist journals
☐ Recommendations from colleagues
☐ Other: [Free text]

**35. Are you aware of patient advocacy organisations for patients with NMOSD or MOGAD?** (Single choice)
( ) Yes
( ) No

**35a. If yes: Which ones?**

[Free text]

**36. Would you like specific information or resources to support you in diagnosing or treating patients with NMOSD and MOGAD?** (Single choice)
( ) Yes
( ) No

**36a. If yes: What and how?**

[Free text]

**37. CASE VIGNETTE (1 of 2)**

- 39-year-old woman
- History of optic neuritis in the right eye (confirmed by ophthalmologist); history of myelitis with residual deficits
- Current symptoms: paraparesis of the legs, bowel dysfunction
- Brain MRI: unremarkable
- Spinal cord MRI: T2-hyperintense lesion at level T1-T8 with contrast enhancement

**37a. Does this patient meet dissemination in space criteria for NMOSD diagnosis (according to the IPND 2015 Criteria)?** (Single choice)
(X) Yes
( ) No

**37b. If the prior optic neuritis were absent, but visual evoked potentials of the right eye were prolonged (despite absence of clinical symptoms in that eye), would the NMOSD diagnostic criteria be met?** (Single choice)
( ) Yes
(X) No

**37c. Based on the above information, does this patient meet the criteria for NMOSD diagnosis (according to the IPND 2015 Criteria) if she is AQP4-IgG-seronegative?** (Single choice)
( ) No, this patient does not have a core clinical characteristic of NMOSD.
( ) No, although this patient presents with a core clinical characteristic of NMOSD, since she is AQP4-IgG-seronegative.
(X) Yes, provided there is no better explanation, because she has a core clinical characteristic of NMOSD, a history of other core clinical characteristics, and the required supporting MRI findings.
( ) No, this patient presents with a core clinical characteristic of NMOSD, but meets neither dissemination in space nor dissemination in time criteria and therefore does not have NMOSD.

**37. CASE VIGNETTE (2 of 2)**

- 28-year-old woman
- History of bilateral optic neuritis without residual deficits
- Current symptoms: recurrent visual deterioration in the right eye, pronounced pain with eye movement
- Brain MRI: bilateral longitudinally extensive T2-hyperintense optic nerves, otherwise unremarkable
- Spinal cord MRI: unremarkable

**37a. Do you test this patient for MOG antibodies?** (Single choice)
( ) Yes, because bilateral optic neuritis excludes the diagnosis of multiple sclerosis.
( ) No, the absence of residual deficits from prior optic neuritis suggests the diagnosis of multiple sclerosis.
( ) No, this clinical presentation is more typical of NMOSD and I only test for AQP4 antibodies.
(X) Yes, in combination with testing for AQP4 antibodies.
( ) No, because a diagnosis of MOGAD could not be established due to the absence of dissemination in space.

**37b. Does this patient meet the current diagnostic criteria for MOGAD if the MOG-IgG titre is only borderline positive with 1:32?** (Single choice)
( ) No, because she has only one core clinical event of MOGAD.
( ) No, although she has a core clinical event of MOGAD, since she lacks supporting clinical or radiologic features.
(X) Yes, provided she is AQP4-IgG-seronegative and alternative diagnoses have been excluded, because she has a core clinical characteristic of MOGAD with required supporting MRI findings.
( ) No, she demonstrates a core clinical event with supporting MRI findings of NMOSD, so MOGAD diagnostic criteria should not be applied.
